# Identification and treatment of persistent small airway dysfunction in paediatric patients with asthma: a retrospective cohort study

**DOI:** 10.1186/s12890-024-02907-z

**Published:** 2024-02-23

**Authors:** Lulu Zhang, Zhou Fu, Hua Deng, Qin Xie, Wenjie Wu

**Affiliations:** 1 Department of Respiratory Medicine, Children’s Hospital of Chongqing Medical University, National Clinical Research Center for Child Health and Disorders, Ministry of Education Key Laboratory of Child Development and Disorders, Chongqing Key Laboratory of Pediatrics, NO.136, Zhongshan Second Road, 400014 Chongqing, China; 2 Department of Pediatric Internal Medicine, Chongqing Youyoubaobei Women and Children’s Hospital, NO.999, Jiarong Road, 401122 Chongqing, China

**Keywords:** Paediatric asthma, Persistent small airway dysfunction, Identification, Treatment, Risk factors

## Abstract

**Background:**

Asthma is a common respiratory disease. In asthma, the small airways have more intensive inflammation and prominent airway remodelling, compared to the central airways. We aimed to investigate the predictive value of risk factors and the fractional concentration of exhaled nitric oxide (FeNO) for persistent small airway dysfunction (p-SAD), and compare the effects of different treatment modalities.

**Methods:**

This retrospective cohort study included 248 children with asthma (aged 4–11 years). Binary logistic regression was used to analyse the risk factors for p-SAD. Correlations among FEV_1_/FVC, small airway function parameters, and FeNO levels in patients with asthma were analysed using Spearman’s rank correlation. The receiver operating characteristic curve and the Delong test were used to analyse the predictive value of FeNO for p-SAD. Differences in the treatment effects of inhaled corticosteroids (ICS) and ICS with a long-acting beta-agonist (ICS/LABA) on p-SAD were analysed using Fisher’s exact test.

**Results:**

Asthmatic children with older age of receiving the regular treatment (OR 1.782, 95% CI 1.082–2.935), with younger age at the time of onset of suspected asthma symptoms (OR 0.602, 95% CI 0.365–0.993), with longer duration of using ICS or ICS/LABA (OR 1.642, 95% CI 1.170–2.305) and with worse asthma control (OR 3.893, 95% CI 1.699–8.922) had increased risk for p-SAD. Significant negative correlations of small airway function parameters with FeNO at a 200 mL/s flow rate (FeNO_200_), and the concentration of nitric oxide in the alveolar or acinar region (CaNO) were observed. The areas under the curve of FeNO_200_ (cut-off:10.5ppb), CaNO (cut-off:5.1ppb), and FeNO_200_ combined with CaNO were 0.743, 0.697, and 0.750, respectively, for asthma with p-SAD. After using ICS or ICS/LABA, switching to ICS/LABA was easier than continuing with ICS to improve small airway dysfunction (SAD) in the 8th month.

**Conclusions:**

Paediatric asthma with p-SAD is associated with older age at receiving regular treatment, younger age at the time of onset of suspected asthma symptoms, longer duration of using ICS or ICS/LABA, worse asthma control, and higher FeNO_200_ and CaNO levels, all of which can be combined with small airway function indicators to distinguish p-SAD from asthma. ICS/LABA improves SAD better than ICS alone.

## Background

Asthma is a heterogeneous and chronic respiratory disease characterised by airway inflammation, airway hyperresponsiveness, and impaired pulmonary function [1]. Central and peripheral airway obstruction is commonly observed in patients with asthma [2]. Small airway dysfunction (SAD) has attracted widespread attention in asthma research. Small airways are defined as airways with internal diameters less than 2 mm in adults [3]. Compared to the central airways, the small airways are more intensively inflamed and undergo a more prominent airway remodelling in asthma [4].

Epidemiological studies have shown that 25–33% of children with asthma have SAD [5], which is particularly prevalent in patients with severe and difficult-to-treat asthma [6]. A longitudinal cohort study indicated that SAD increases the risk of increased asthma symptoms and exacerbations [7]. SAD has been linked to more severe bronchial hyperresponsiveness, worse asthma control, and a greater number of exacerbations [8, 9]. However, SAD was recently detected in children with mild asthma [10], and SAD may even be present in the absence of symptoms as well as in patients with normal spirometry [11]. Some children with asthma and SAD exhibit transient symptoms due to environmental infections or poor asthma control that disappear quickly after treatment. However, other children may progress to persistent small airway dysfunction (p-SAD) with poor prognosis. In clinical practice, pulmonary function tests are routinely performed to evaluate small airway function. Multiple methods should be used in conjunction to identify p-SAD at an earlier stage in patients with asthma. However, the guiding value of demographics, clinical characteristics, and fractional concentration of exhaled nitric oxide (FeNO) in predicting p-SAD in asthma is unknown. There is growing recognition that the role of small airways in asthma may be a target for optimal disease control. Treatment of small airway obstruction effectively reduces asthma symptoms, controls airway inflammation, and decreases the incidence of acute exacerbations [12]. A personalised approach to treating asthma with SAD includes increasing the dose of inhaled corticosteroids (ICS) or combining ICS with a long-acting beta-agonists (ICS/LABA) [13]. However, an effective regimen for treating paediatric patients with asthma and p-SAD has not yet been developed.

Therefore, in this study, we aimed to reveal the clinical characteristics of paediatric asthma with p-SAD, to investigate the guiding value of the risk factors, and FeNO in the prediction of p-SAD in asthma. Moreover, we explored better treatment plans for asthma with p-SAD. Based on this research, we hope to provide a strong basis for the identification and treatment of p-SAD in paediatric patients with asthma, which would aid in improving their prognosis.

## Methods

### Study population

Paediatric patients diagnosed with asthma at the Respiratory Specialist Clinic of Children’s Hospital of Chongqing Medical University and Chongqing Youyoubaobei Women and Children’s Hospital between January 2019 and September 2021 were included in our study.

The inclusion criteria were as follows: (1) patients aged between 4 and 11 years; (2) those diagnosed with bronchial asthma in accordance with the Guideline for the Diagnosis and Optimal Management of Asthma in Children [14]; (3) patients who received treatment with ICS or ICS/LABA for more than 6 months; and (4) those who were followed up for at least 1 year during which pulmonary function tests were performed more than four times every 2 months or longer.

The exclusion criteria for the study were as follows: (1) patients with other diseases, such as mental retardation, cardiac anomalies, congenital malformations, other diseases of the lungs/airways, kidney diseases, immunodeficiency diseases, and other diseases requiring hormonal treatment; (2) those who were not compliant with their asthma medication treatment; and (3) patients who were not followed up regularly or those who were lost to follow-up.

### Study design and data collection

**Fig. 1:**
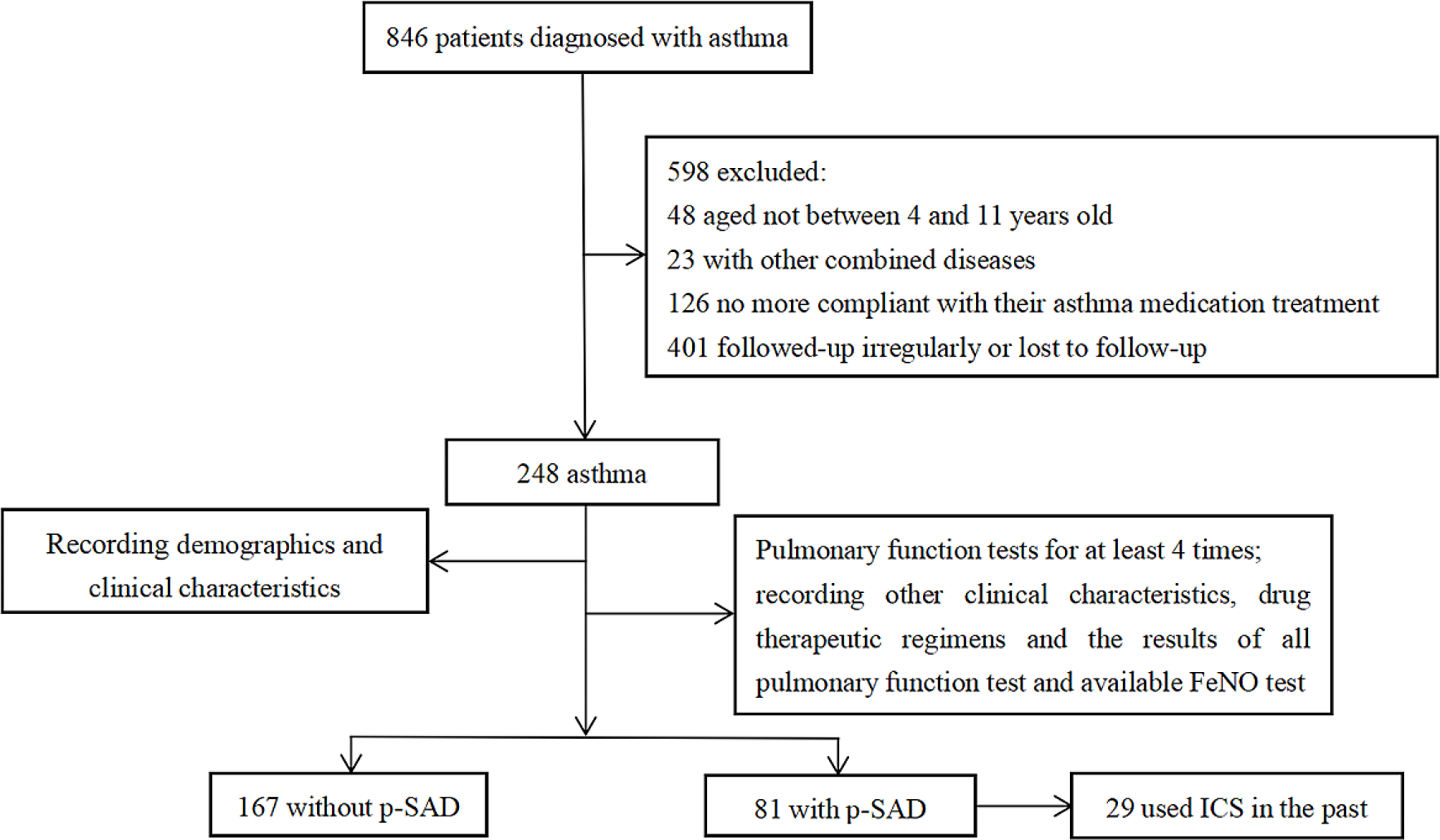
Patient screen and follow-up. FeNO, fractional concentration of exhaled nitric oxide; ICS, inhaled corticosteroids; ICS/LABA, combined ICS with a long-acting beta-agonist; p-SAD, persistent small airway dysfunction

The initial sample size of the research cohort of patients with asthma was 846, of whom, 598 were excluded. In total, 248 patients with asthma were included in this study. The demographics and clinical characteristics of the 248 study participants were analysed. We also collected data regarding other clinical characteristics, drug therapeutic regimens, results of all pulmonary function tests, and available FeNO tests of the 248 children with asthma. SAD was defined as < 65% of any two of the following parameters: forced expiratory flow between 25 and 75% of forced vital capacity (FEF_25 − 75_)% pred, forced expiratory flow at 50% of forced vital capacity (FEF_50_)% pred, and forced expiratory flow at 75% of forced vital capacity (FEF_75_)% pred measurements [15, 16]. SAD with the abovementioned pulmonary function test results for two consecutive times was designated as p-SAD. During the follow-up period of at least 1 year (14–25 months), 81 children with asthma developed p-SAD, the diagnosis of which was based on the results of pulmonary function tests. Therefore, we divided the children with asthma into two groups: an asthma with p-SAD group comprising 81 participants and an asthma without p-SAD group comprising 167 patients. We continued to analyse the treatment and improvement of SAD in patients with p-SAD in the ensuing 8 months after the appearance of p-SAD, including patients who continuously used ICS in the past. The study protocol is illustrated in Fig. 1.

### Assessments

Asthma control level: Based on the responsiveness to treatment, the patients were classified into well-controlled, partly-controlled (met 1–2 assessing projects), and uncontrolled (met ≥ 3 assessing projects) groups. Specific symptom control level grading was based on the Guideline for the Diagnosis and Optimal Management of Asthma in Children (2016) [14].

Pulmonary function test: We used MasterScreen Pead (EAGER Company, Germany) to perform the pulmonary function tests. Spirometry along with bronchial provocation or bronchodilation tests were performed as recommended by the American Thoracic Society (ATS) [17]. Short-acting beta-agonists should be stopped at least 8–12 h, short -acting muscarinic-agonists should be stopped at least 24 h, LABA/long-acting muscarinic-agonists should be stopped at least 24–48 h, leukotriene receptor antagonists should be discontinued at least 48 h and ICS should be discontinued at least 24 h before performing spirometry or either a bronchial provocation test (using methacholine) or a bronchodilator test (using salbutamol). The instrument automatically calculates and obtains the measurement parameters. At least three attempts were required and all tests were recorded. Finally, two well-trained technicians reviewed the flow-volume and volume-time tracings and selected the best results to save. Reference values of spirometry parameters for children were adjusted for age, height, weight and sex [18]. The indicators included forced vital capacity (FVC), forced expiratory volume in the first second (FEV_1_), FEV_1_/FVC, (FEF_75_)% pred, (FEF_50_)% pred, FEF(_25−75_)% pred.

FeNO test: FeNO was measured according to the standard procedures recommended by the ATS and the European Respiratory Society (ERS) [19]. A Nano Coulomb Nitric Oxide Analyzer (Shangwo Biotechnology Company, Wuxi, China) was used for the tests. Participants were informed to inhale nitric oxide (NO)-free air and exhaled via a mouthpiece at a constant flow rate of 50 mL/s for at least 4 s and 200 mL/s for at least 2 s. Then, FeNO and the concentration of nitric oxide in the gas phase of the alveolar or acinar region (CaNO) were automatically calculated by the analyser. CaNO was calculated using a two-compartment model based on FeNO at different flow rates (50 and 200 mL/s). Three tests were repeated, and the average values of FeNO at a 50 mL/s flow rate (FeNO_50_), FeNO at a 200 mL/s flow rate (FeNO_200_), and CaNO were used for subsequent analyses.

### Statistical analysis

Continuous data with normal distribution are presented as mean ± standard deviation (SD), and non-normally distributed data are presented as median and interquartile range (IQR). All dichotomous data are presented as numbers and percentages [n (%)]. For group comparisons, Student’s t-test or one-way analysis of variance (ANOVA) was used for normally distributed continuous variables, Mann-Whitney U-test or Kruskal-Wallis H-test was used for non-normally distributed continuous variables, and the Chi-square test or Fisher’s exact test was used for dichotomous variables. To test the correlations between two continuous variables, we used Pearson’s correlation for normally distributed data and Spearman’s rank correlation for non-normally distributed data. To investigate the risk factors, independent variables were included in a binary logistic regression model using the enter method. The odd ratio (OR) with 95% confidence intervals (CI) obtained in the regression analysis were calculated. The optimal cut-off points were determined using receiver operating characteristic (ROC) analysis. Comparing the area under the curve (AUC) of ROC was performed using the Delong test. Data were analysed using SPSS software for Windows (version 27) and the R programming language, and two-tailed *p*-values < 0.05 were considered statistically significant.

## Results

### Demographics and clinical characteristics of the study participants

Demographics and clinical characteristics were compared between patients with and without p-SAD (Table 1). Among the 248 participants with asthma, 167 belonged to the asthma group without p-SAD and 81 to the asthma group with p-SAD. Compared to patients without p-SAD, those with p-SAD had a significantly higher ratio of a history of passive smoking (35.8% vs. 22.8%, *P* = 0.03), younger age at the time of onset of suspected asthma symptoms (5.00 years old vs. 6.00 years old, *P* = 0.047), longer duration of asthma (4.50 years vs. 3.50 years, *P* = 0.007), and longer duration of using ICS or ICS/LABA (3.00 years vs. 2.50 years, *P* < 0.001). Patients with p-SAD had partly-controlled (20.99%) or well-controlled (7.19%) asthma, whereas most of the patients without p-SAD had well-controlled (92.81%) or partly-controlled (79.01%) asthma (*P* = 0.002). There were no significant differences between the two groups in terms of age of receiving the regular treatment, sex, BMI, birth weight, breastfeeding, history of antibiotic usage in the first year, family history of asthma, allergens, allergic rhinitis, eczema, time taken for asthma to be well controlled, and age at which the diagnosis of asthma was made (*P* > 0.05).

**Table 1:** Demographics and clinical characteristics of patients with and without p-SAD

Item	Asthma without p-SAD (*n* = 167)	Asthma with p-SAD (*n* = 81)	Z/χ^2^	*P*
Age of receiving the regular treatment (years old)	7.00(6.00,8.50)	7.50(6.50,9.00)	-1.837	0.067
Male	111(66.5)	55(67.9)	0.051	0.822
BMI (kg/m^2^)	16.50(15.20,17.80)	16.90(14.95,18.90)	-0.061	0.951
Birth weight (kg)	3.20(3.00,3.40)	3.20(3.00,3.45)	0	1
Breastfeeding (months)	7.00(3.00,11.00)	6.00(4.50,10.00)	-0.538	0.591
History of antibiotic usage in the first year	50(29.9)	25(30.8)	0.022	0.882
History of passive smoking	38(22.8)	29(35.8)	4.71	0.03
Family history of asthma	25(15.0)	20(24.7)	3.471	0.062
Allergens	119(71.3)	55(67.9)	0.293	0.588
Allergic rhinitis	138(82.6)	64(79.0)	0.474	0.491
Eczema	37(22.2)	15(18.5)	0.435	0.509
Asthma control				
Well-controlled	155(92.81)	64(79.01)	10.062	0.002
Partly-controlled	12(7.19)	17(20.99)		
Time taken for asthma to reach well controlled (years)	1.00(1.00,1.00)	1.00(1.00,2.00)	-1.002	0.316
Age at the time of onset of suspected asthma symptoms (years old)	6.00(4.50,7.00)	5.00(3.50,7.00)	-1.989	0.047
Duration of asthma (years)	3.50(3.00,5.00)	4.50(3.00,7.25)	-2.688	0.007
Age at which the diagnosis of asthma was made (years old)	6.50(6.00,8.00)	7.00(5.75,8.25)	-0.143	0.886
Duration of using ICS or ICS/LABA (years)	2.50(2.00,3.00)	3.00(2.00,3.50)	-3.724	< 0.001

Data were presented as median (25%, 75% IQR) or number (%); the difference between groups was analyzed by Mann-Whitney U-test or chi-squared test. BMI, body mass index; p-SAD, persistent small airway dysfunction; ICS, inhaled corticosteroids; LABA, long-acting beta-agonist

### Binary logistic regression analysis to identify risk factors for asthma with p-SAD

Possible factors influencing asthma with p-SAD (*P* < 0.1) were used as independent variables. The presence of p-SAD was used as the dependent variable. These factors were included in the binary logistic regression analysis (Table 2). The analysis results showed that asthmatic children with older age of receiving the regular treatment (OR 1.782, 95% CI 1.082–2.935, *P* = 0.023), younger age at the time of onset of suspected asthma symptoms (OR 0.602, 95% CI 0.365–0.993, *P* = 0.047), longer duration of using ICS or ICS/LABA (OR 1.642, 95% CI 1.170–2.305, *P* = 0.004) and worse asthma control (OR 3.893, 95% CI 1.699–8.922, *P* = 0.001) indicated the possibility of occurrence of p-SAD.

**Table 2:** Binary logistic regression analysis of risk factors for asthma with p-SAD

Variables	OR (95% CI)	*P*
Age of receiving the regular treatment	1.782(1.082–2.935)	0.023
History of passive smoking	1.733(0.921–3.260)	0.088
Family history of asthma	1.693(0.824–3.481)	0.152
Asthma control (Partly-controlled)	3.893(1.699–8.922)	0.001
Age at the time of onset of suspected asthma symptoms	0.602(0.365–0.993)	0.047
Duration of asthma	0.664(0.399–1.104)	0.114
Duration of using ICS or ICS/LABA	1.642(1.170–2.305)	0.004

ICS, inhaled corticosteroids; LABA, long-acting beta-agonist; OR, Odd Ratio; CI, confidence intervals

### Correlations among FEV_1_/FVC, small airway function parameters, and FeNO levels in patients with asthma

During the follow-up period, some patients with asthma underwent FeNO testing. Significant positive correlations of FEV_1_/FVC with (FEF_75_)% pred (*r* = 0.754, *P* < 0.001), (FEF_50_)% pred (*r* = 0.895, *P* < 0.001), and (FEF_25 − 75_)% pred (*r* = 0.930, *P* < 0.001) were observed. We found negative correlations between FEV_1_/FVC and FeNO_50_ (*r*=-0.262, *P* = 0.018). Negative correlations of FeNO_200_ with (FEF_75_)% pred (*r*=-0.456, *P* < 0.001), (FEF_50_)% pred (*r*=-0.353, *P* = 0.003), and (FEF_25 − 75_)% pred (*r*=-0.401, *P* < 0.001) were also found. Furthermore, negative correlations of CaNO with (FEF_75_)% pred (*r*=-0.297, *P* = 0.015), (FEF_50_)% pred (*r*=-0.281, *P* = 0.021), and (FEF_25 − 75_)% pred (*r*=-0.267, *P* = 0.029) were observed.

### ROC curve analysis of FeNO levels for predicting the diagnosis of asthma with p-SAD

**Fig. 2:**
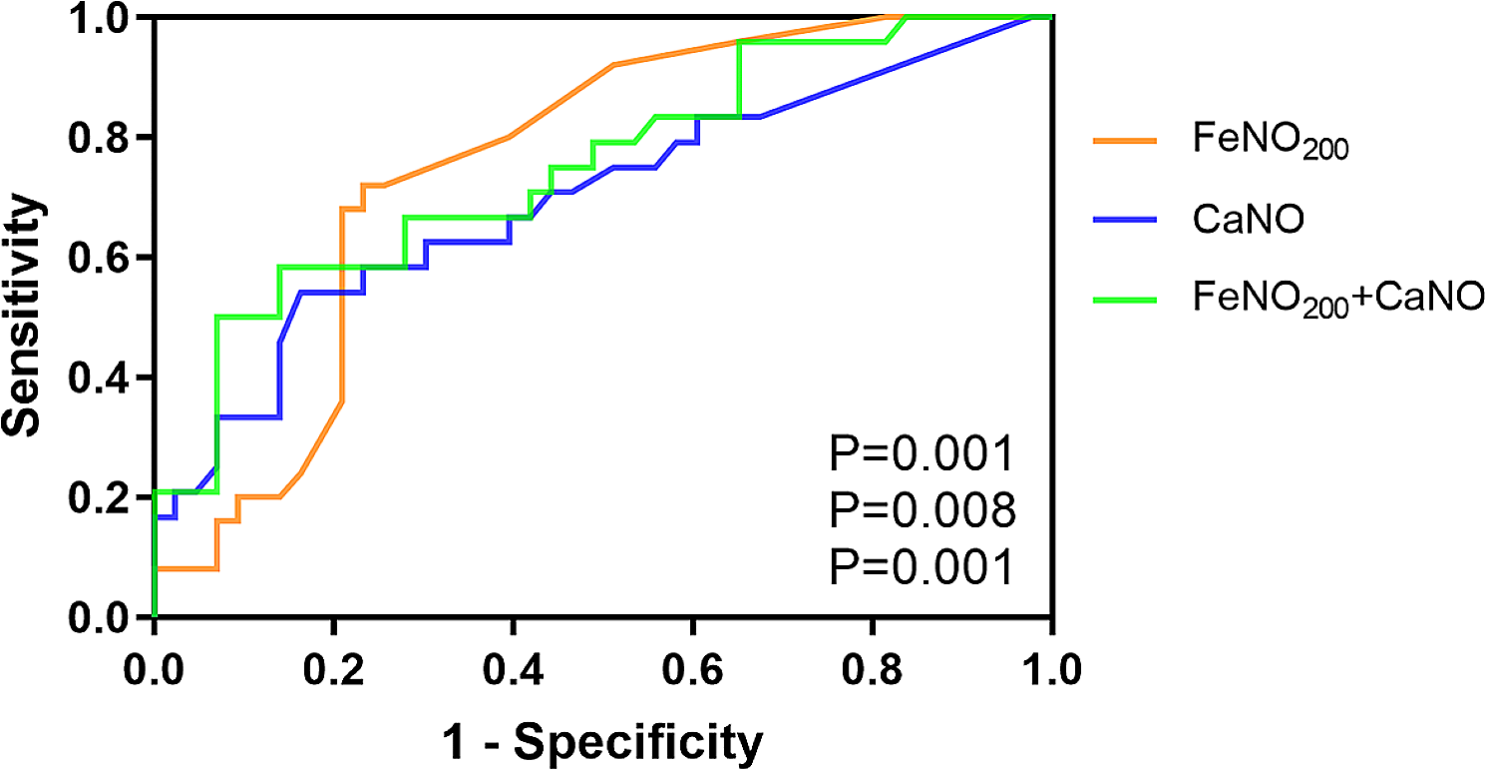
ROC curve analysis of FeNO_200_ or CaNO alone and in combination for predicting the diagnosis of asthma with p-SAD. FeNO_200_, fractional concentration of exhaled nitric oxide at a 200 mL/s flow rate; CaNO, concentration of nitric oxide of the alveolar or acinar region

For patients with p-SAD, the values of FeNO_200_ and CaNO were 15.00 and 5.20 ppb, respectively. These values were significantly higher than that of patients without p-SAD (*P* < 0.001, *P* = 0.007). However, the value of FeNO_50_ did not significantly differ between the two groups (*P* = 0.285). The ROC analysis of asthma with p-SAD showed that the AUC was 0.743 (95% CI:0.624–0.861, *P* = 0.001) for FeNO_200_ alone, 0.697 (95% CI:0.561–0.834, *P* = 0.008) for CaNO alone, and 0.750 (95% CI:0.627–0.873, *P* = 0.001) for FeNO_200_ combined with CaNO (Fig. 2). The sensitivity and specificity of FeNO_200_ in predicting SAD were 70.8% and 76.7%, respectively, at a cut-off point of 10.5 ppb. The sensitivity and specificity of CaNO in predicting SAD were 54.2% and 83.7%, respectively, at a cut-off point of 5.1 ppb. The sensitivity and specificity of FeNO_200_ combined with CaNO in predicting SAD was 58.3% and 86%, respectively. There were no significant differences between the AUCs of CaNO and FeNO_200_, CaNO and FeNO_200_ combined with CaNO, FeNO_200_ and FeNO_200_ combined with CaNO.

### Improvement of SAD in asthma with p-SAD

During the follow-up period of at least 1 year (14–25 months), 81 children with asthma developed p-SAD. This diagnosis was made based on two consecutive pulmonary function tests. We continued to record their drug therapeutic regimens and therapeutic effects in the 4th and 8th month after p-SAD onset (Table 3). In patients who received continuous treatment with ICS, the small airway function of 5 and 7 patients returned to normal in the 4th and 8th month, respectively (*P* = 0.049 and *P* = 0.001). In patients who continuously received ICS/LABA treatment, the small airway function of 13 and 30 patients returned to normal in the 4th (*P* < 0.001) and 8th month, respectively (*P* < 0.001).

According to the Global Initiative for Asthma Strategy 2021 and clinical experience [1], some patients with p-SAD who continuously used ICS adjusted their drug treatment plans. We separately analysed the specific drug adjustments made. On comparing the number of patients whose small airway function returned to normal after continuing the use of ICS or those who switched to ICS/LABA (Table 4), no significant differences were observed in the 4th month (*P* = 0.108). However, in the 8th month, the number of patients whose small airway function returned to normal comprised more patients who switched to ICS/LABA than those who continued using ICS (*P* = 0.029).

**Table 3:** Improvement of SAD after continuing to using ICS or ICS/LABA

Item 1	Item 2	Asthma with SAD	Asthma without SAD	χ^2^	*P*
Using ICS					
	In the 4th month	17(77.3)	5(22.7)	/	0.049
	In the 8th month	9(56.3)	7(43.7)	/	0.001
Using ICS/LABA					
	In the 4th month	46(78.0)	13(22.0)	/	< 0.001
	In the 8th month	35(53.8)	30(46.2)	/	< 0.001

Data were presented as number (%); the difference between groups was analyzed by the fisher exact test

ICS, inhaled corticosteroids; LABA, long-acting beta-agonist; SAD, small airway dysfunction

**Table 4:** Improvement of SAD in patients who continuously used ICS in the past

Item 1	Item 2	Asthma with SAD	Asthma without SAD	χ^2^	Unilateral P
In the 4th month					
	Using ICS	17(77.3)	5(22.7)	/	0.108
	Using ICS/LABA	3(42.9)	4(57.1)		
In the 8th month					
	Using ICS	9(56.3)	7(43.7)	/	0.029
	Using ICS/LABA	2(15.4)	11(84.6)		

Data were presented as number (%); the difference between groups was analyzed by the fisher exact test

ICS, inhaled corticosteroids; LABA, long-acting beta-agonist; SAD, small airway dysfunction

## Discussion

In this study, we combined significant risk factors, pulmonary function and FeNO levels to distinguish p-SAD from asthma. Moreover, we explored better treatment plans for asthma with p-SAD. Asthmatic children with older age of receiving the regular treatment, younger age at the time of onset of suspected asthma symptoms, longer duration of using ICS or ICS/LABA, and worse asthma control indicated the possibility of occurrence of p-SAD. There is a negative correlation between small airway function parameters and FeNO_200_ and CaNO levels. However, because of its relatively low sensitivities and specificities, the use of FeNO as a diagnostic tool for SAD is controversial. In some children, we found that the treatment effect of ICS/LABA was better than that of ICS alone in improving SAD. Based on the above findings, we hope to provide a strong basis for the identification and treatment of p-SAD in paediatric patients with asthma.

Numerous physiological and imaging techniques have been used to assess small airway function including spirometry, impulse oscillometry (IOS), multiple breath washout (MBW), exhaled NO (eNO), hyperpolarised magnetic resonance imaging, high resolution computed tomography and nuclear medicine [10, 20, 21]. Currently, there is no gold standard method for clinically assessing SAD. International asthma guidelines suggest spirometry as the method of choice for assessing lung function [1]. Therefore, we used lung function parameters to detect SAD, which is the most acceptable and feasible method in routine clinical practice. FEV_1_ and FEV_1_/ FVC mainly represent the larger airways, whereas (FEF_25–75_)% pred, (FEF_50_)% pred, and (FEF_75_)% pred reflect the small airway function. The (FEF_75_)% pred is a more sensitive parameter reflecting SAD in asthma [22]. We defined the SAD of lung function parameters for two or more consecutive measurements as p-SAD. Our findings suggest that small airway parameters are consistent with FEV_1_/ FVC when evaluating airway obstruction. Moreover, it can be used as a sensitive measurement for assessing asthma in children in the remission stage, where FEV_1_/ FVC remains within the normal range until the irreversible small-airway disease phase occurs. Therefore, monitoring of small airway function should be emphasised during the remission stage of asthma, and small airway lesions should not be ignored when evaluating the long-term prognosis of children with asthma. However, there are limitations of pulmonary function tests in assessing asthma, including poor cooperation of children, operational variation by physicians, and inaccurate assessment due to insufficient number of tests. Therefore, we combined the pulmonary function test with clinical risk factors and the FeNO test.

Some studies of adults found that advanced age, female sex, passive smoking, age at asthma diagnosis and so on have been significantly associated with the risk of SAD with asthma [11, 15, 23]. Although, there is an increasing number of studies on small airway dysfunction in paediatric asthma, little is known about the clinical risk factors for SAD. In our study, older age of receiving the regular treatment, younger age at the time of onset of suspected asthma symptoms, longer duration of using ICS or ICS/LABA, and worse asthma control were risk factors for asthma appearing in p-SAD. Asthma in children usually occurs before 3 years of age, and persistent impairment of lung function may occur in preschool children [14]. Some children develop asthma symptoms early; however, some doctors with low awareness of asthma treat recurrent wheezing as common cold. In addition, some children had been diagnosed with asthma and some parents had low medical compliance with treatment, resulting in delayed treatment. A longer duration of using ICS or ICS/LABA indicates poor asthma control. Dysfunction caused by persistent small airway inflammation in the periphery is closely linked to the degree of asthma control [24]. Severe SAD may represent a unique eosinophilic asthma phenotype [25], and patients with asthma and SAD have increased numbers of IgA + memory B cells [26]. These children may have persistent abnormal lung function and airway inflammation, resulting in p-SAD. Huang et al. [27] demonstrated that most children with well-controlled asthma continued to have airway hyperresponsiveness and poor small airway function. Therefore, regular and continuous monitoring of asthma in children is necessary.

FeNO is a useful non-invasive biomarker reflecting Th2-driven lung airway and alveolar inflammation, which is usually mediated by eosinophils [25]. FeNO_50_ can reflect inflammation, mainly in the large central airways. FeNO_200_ reflects inflammation dynamics of the small airways or lung parenchyma. CaNO may be valuable for evaluating inflammation in the small airways or lung parenchyma in both airway and interstitial lung disease [28]. Our results showed that FeNO_50_ was negatively correlated with large airway function, whereas FeNO_200_ and CaNO were negatively associated with small airway function. These correlations of CaNO levels with airway function were consistent with previous reports, suggesting that alveolar NO is a marker of peripheral airway dysfunction [29–31]. Our results revealed that the values of FeNO_200_ and CaNO in patients with asthma of p-SAD were significantly higher than those in patients with asthma but without p-SAD, which was similar to the results of the following studies. Mahut et al. [32] showed that CaNO levels were significantly higher in recently symptomatic asthmatic children than in asthmatic patients without symptoms, possibly reflecting deep lung inflammatory cell recruitment. Scichilone et al. [33] found that in adults, NO contribution from small airways (CalvNO) was significantly higher in patients with uncontrolled asthma than in those with controlled/partially controlled asthma. These findings support the characterisation of SAD by elevated CaNO levels. Some studies recommend adjustments of CalvNO for the trumpet model and axial diffusion (TMAD). In a large cohort study of 410 participants [34] aged 10–35 years with asthma, both FeNO and unadjusted CalvNO levels were related to asthma symptoms, lung function and bronchial responsiveness. However, no associations between TMAD-adjusted CalvNO and asthma characteristics were found, raising the questions of overadjustment. Further studies assessing axial diffusion in patients with asthma and the validity of the proposed adjustment algorithms are warranted.

Glucocorticoids and β_2_ receptor agonists are essential for the management of patients with asthma [35]. In our study, both ICS and ICS/LABA significantly improved SAD in the 4th and 8th month. The difference in the improvement of SAD between the two medication regimens was significant in the 8th month. Thus, we concluded that ICS and ICS/LABA improved SAD, and that concurrent use of ICS and LABA was better. Despite treatment with a high dose of ICS/LABA in our study, several patients still developed SAD. This may have been because the ICS used by the children in our study was fluticasone propionate, which is a drug with a smaller molecular weight to choose from. Currently, traditional ICS transported by most inhalers [mean mass aerodynamic diameter (MMAD)] ≥ 2 μm and < 5 μm] do not sufficiently reach the small airways, whereas the extra-fine ICS (MMAD <2 μm) transported by hydrogen fluorine alkane (HFA) have a higher pulmonary deposition and can better penetrate the small airways. Moreover, they also obviously reduce the daily ICS dose [36]. Vos et al. [37] reported that an extra-fine inhaler may improve SAD and clinical outcomes and asthma control in patients with asthma. In addition, this matter may also be related to the short follow-up duration. Therefore, further studies are needed to determine whether extra-fine-particle inhalers or long-term treatment will improve small airway function and prognosis in patients with p-SAD.

Our study has some limitations that need to be taken into consideration. First, internationally, spirometry indices are usually expressed as z-scores (number of standard deviations by which the measurement differs from the mean predicted value) using the prediction equations from the Global Lung Function Initiative 2012 (GLI-2012). However, we used the measured values/expected values of spirometry indices to evaluate pulmonary function, a method commonly used in China based on statistical results of several large-sample studies. Second, an international multiple-flow analysis of FeNO is needed to differentiate between proximal airway and alveolar NO. However, in our study, CaNO was calculated using a two-compartment model based on FeNO at different flow rates (50 and 200 mL/s). Therefore, in a subsequent study, we hope to utilize the latest international standards and methods. Even, we can widely use some other advanced techniques, such as IOS and MBW. Finally, this was a retrospective study in which the data might have been partially biased, and the sample size was limited. Prospective research with a larger sample size is needed for further analysis to provide more evidence in support of clinical practice guidelines.

## Conclusions

In conclusion, older age of receiving regular treatment, younger age at the time of onset of suspected asthma symptoms, longer duration of using ICS or ICS/LABA, and worse asthma control might identify children with asthma at a risk for p-SAD. There is a negative correlation between small airway function parameters and FeNO_200_ and CaNO, supporting the characterisation of SAD by elevated CaNO levels. ICS/LABA improves SAD better than ICS in some children with asthma, and an extra-fine inhaler may better penetrate the small airways. This study provides a basis for the identification and treatment of p-SAD in paediatric patients with asthma. The findings of our study may aid in improving the prognosis of children with asthma.

## Data Availability

The datasets generated and/or analyzed during the current study are not publicly available but are available from the corresponding author on reasonable request.

## Abbreviations

SAD: Small airway dysfunction
p-SAD: Persistent small airway dysfunction
ICS: Inhaled corticosteroids
ICS/LABA: Combine ICS with a long-acting beta-agonists
FEF_25 − 75_: Forced expiratory flow between 25 and 75% of forced vital capacity
FEF_50_: Forced expiratory flow at 50% of forced vital capacity
FEF_75_: Forced expiratory flow at 75% of forced vital capacity
FVC: Forced vital capacity
FEV_1_: Forced expiratory volume in the first second
FeNO: Fractional concentration of exhaled nitric oxide
FeNO_50_: FeNO at a 50mL/s flow rate
FeNO_200_: FeNO at a 200mL/s flow rate
NO: Nitric oxide
eNO: Exhaled NO
CaNO: Concentration of nitric oxide in the gas phase of the alveolar or acinar region
CalvNO: Nitric oxide contribution from small airways
TMAD: Trumpet model and axial diffusion
SD: Mean ± standard deviation
IQR: Interquartile range
ANOVA: One-way analysis of variance
OR: Odd ratio
CI: Confidence intervals
ROC: Receiver operating characteristic
AUC: Area under the curve
ATS: American Thoracic Society
ERS: European Respiratory Society
BMI: Body mass index
IOS: Impulse oscillometry
MBW: Multiple breath washout
MMAD: Mean mass aerodynamic diameter
HFA: Hydrogen fluorine alkane
GLI-2012: Global Lung Function Initiative 2012
